# A new species of *Nemoura* (Plecoptera, Nemouridae) and supplementary description of *Amphinemura cordiformis* from the Nanling Mountains of southern China

**DOI:** 10.3897/zookeys.1039.60144

**Published:** 2021-05-20

**Authors:** Zhao Meng-Yuan, Du Yu-Zhou

**Affiliations:** 1 School of Horticulture and Plant Protection & Institute of Applied Entomology, Yangzhou University, Yangzhou 225009, China Yangzhou University Yangzhou China; 2 Joint International Research Laboratory of Agriculture and Agri-Product Safety, the Ministry of Education, Yangzhou University, Yangzhou 225009, China Yangzhou University Yangzhou China

**Keywords:** Mangshan National Natural Reserve, Maoershan National Natural Reserve, *Nemoura biplatta* sp. nov.

## Abstract

Two species of Nemouridae are described and illustrated from the Nanling Mountains of southern China, including a new species, *Nemoura biplatta***sp. nov.** from Guangxi Zhuang Autonomous Region, and a new regional record species, *Amphinemura cordiformis* Li & Yang, 2006 from Hunan Province. The morphological characteristics of the new species are compared to related taxa and the new images with supplementary description of *A. cordiformis* are also provided.

## Introduction

*Nemoura* Latreille, 1796 and *Amphinemura* Ris, 1902 are the two largest genera of Nemouridae in China. Both of these genera are comprised of approximately 200 valid species known from the Holarctic and Oriental regions (Baumann 1975; Yang et al. 2015; Yang and Li 2018; DeWalt et al. 2020). Currently, more than 40 species of the genus *Nemoura* and nearly 100 species of the genus *Amphinemura* have been recorded from China (Wu 1926, 1938, 1940, 1962, 1973; Sivec 1981; Li and Yang 2005, 2006a, 2006b, 2007a, 2007b, 2008a, 2008b, 2008c, 2008d, 2011; Wang et al. 2006; Du and Wang 2007; Du et al. 2007, 2008; Wang and Du 2008; Li et al. 2012; Ji and Du 2014; Ji et al. 2014; Li et al. 2016, 2018a, 2018b; Chen and Du 2017a, 2017b; Mo et al. 2017, 2019, 2020a, 2020b, 2020c; Qian et al. 2018; Chen 2020).

The Nanling Mountains are located at 24°00'–26°30'N, 110°–116°E and are the boundary of Guangdong province, Guangxi Zhuang Autonomous Region, Hunan province, Jiangxi province, and Fujian province. The mountains are regarded as a priority area for biodiversity conservation, containing 19 wildlife natural reserves, such as Maoershan National Natural Reserve in Guangxi Zhuang Autonomous Region and Mangshan National Natural Reserve in Hunan province (Chen et al. 2015). Historically, two *Nemoura* species were recorded from Maoershan National Natural Reserve, including *N. perforata* Li & Yang, 2006a and *N. cucurbitata* Mo, Wang, Yang & Li, 2020.

Herein, an additional *Nemoura* species, *Nemoura biplatta* sp. nov. from Maoershan National Natural Reserve, is described as new to science and one *Amphinemura* species, *Amphinemura cordiformis* Li & Yang, 2006, is proposed from Guizhou province and it is reported for the first time in Mangshan National Natural Reserve. Detail descriptions, illustrations, and new images of the two species are provided and discussed.

## Materials and methods

All examined specimens were collected by hand or net and preserved in 75% ethanol. Terminalia of adults were examined and illustrated using Keyence VHX-5000 system and final images were prepared using Photoshop CS6. All listed specimens are deposited in the Insect Collection of Yangzhou University (**ICYZU**), Jiangsu Province, China. The new species is named after the morphological characteristics of the terminalia.

## Results

### Nemoura biplatta sp. nov.

Taxon classificationAnimaliaPlecopteraNemouridae

02C2B29A-1324-50AF-8FC2-B331FC06ECFA

http://zoobank.org/813CC274-3443-49FB-8BB9-47394EB48578

**Figure 1.**
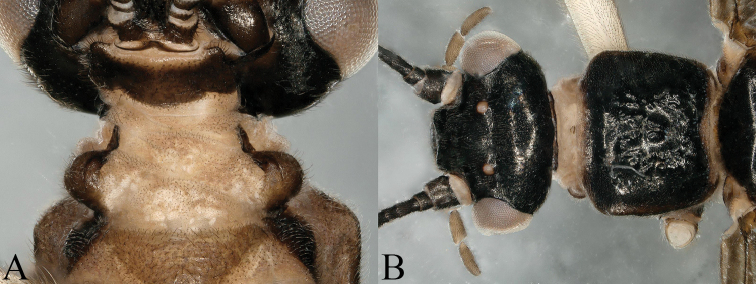
*Nemoura biplatta* sp. nov. male **A** prothorax, ventral view **B** head and pronotum, dorsal view.

**Figure 2.**
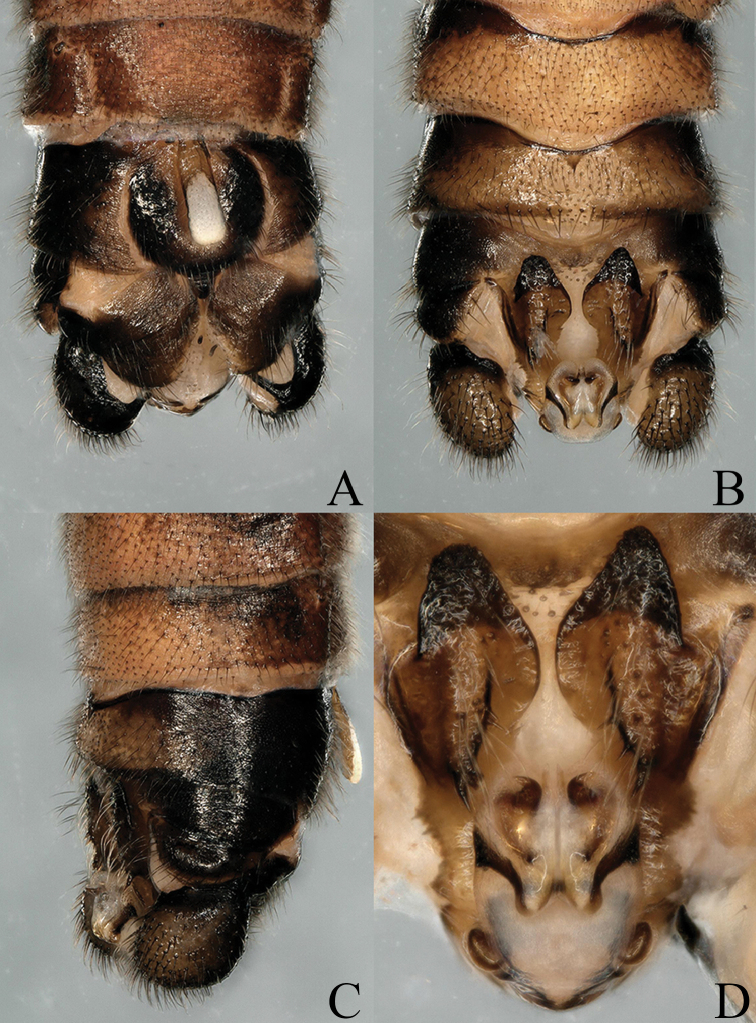
*Nemoura biplatta* sp. nov. male terminalia **A** ventral view **B** dorsal view **C** lateral view **D** epiproct, dorsal view.

**Figure 3.**
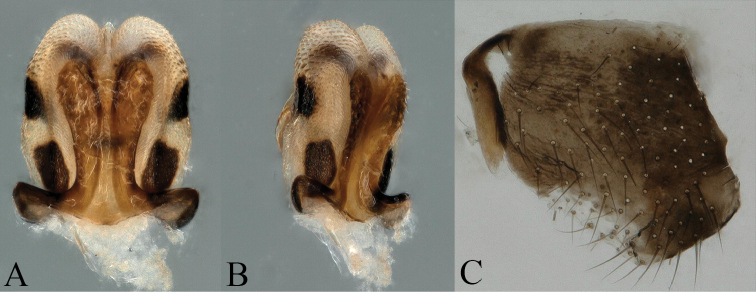
*Nemoura biplatta* sp. nov. male **A** epiproct, ventral view **B** epiproct, lateral view **C** right paraproct, ventral view.

**Figure 4.**
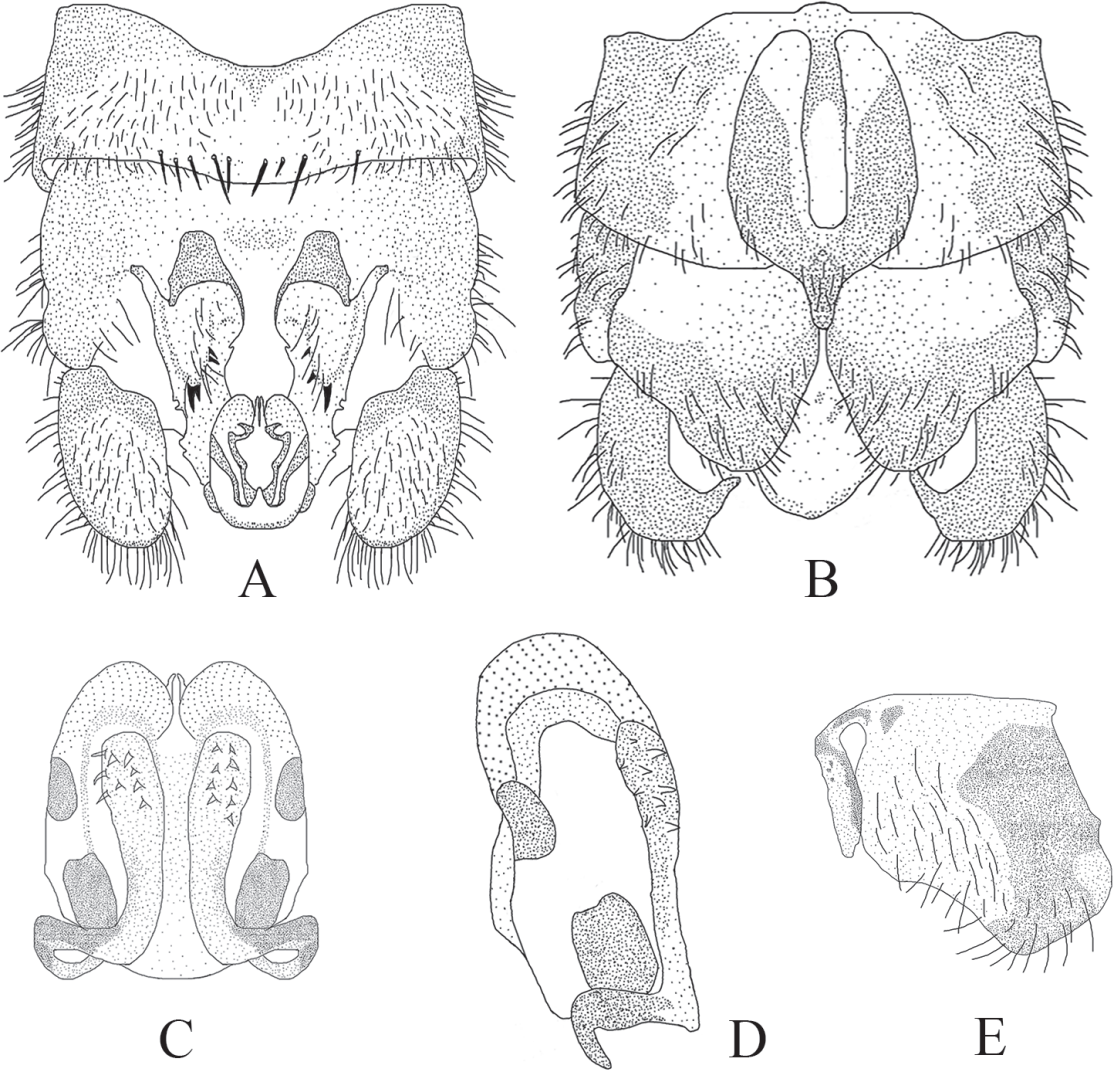
*Nemoura biplatta* sp. nov. male **A** terminalia, dorsal view **B** terminalia, ventral view **C** epiproct, ventral view **D** epiproct, lateral view **E** right paraproct, ventral view.

**Figure 5.**
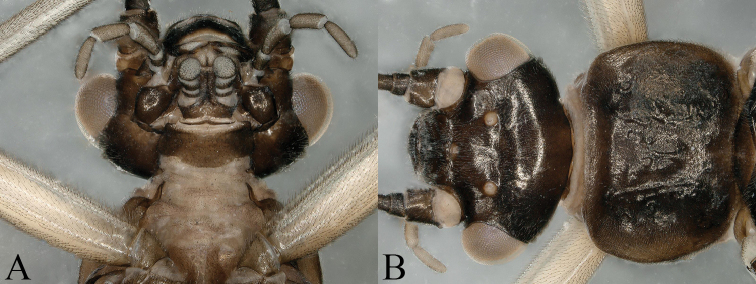
*Nemoura biplatta* sp. nov. female **A** prothorax, ventral view **B** head and pronotum, dorsal view.

**Figure 6.**
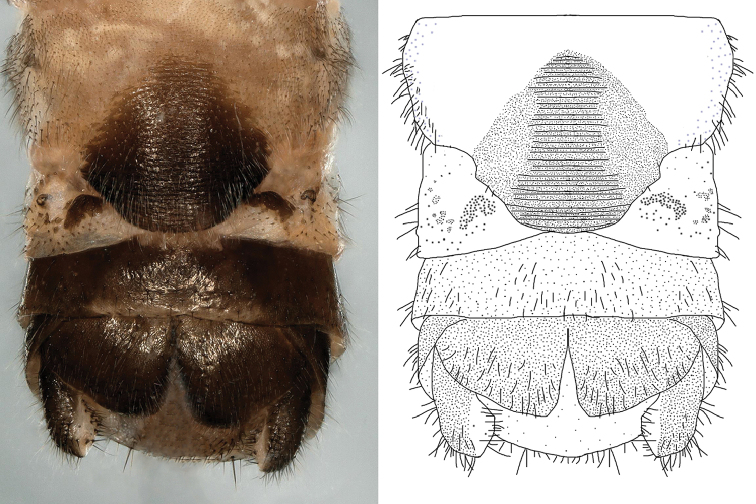
*Nemoura biplatta* sp. nov. female abdomen, ventral view.

**Figure 7.**
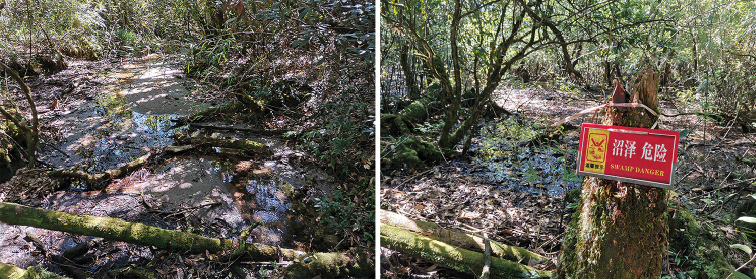
Habitat of *Nemoura biplatta* sp. nov. in China, Guangxi Zhuang Autonomous Region (Maoershan National Natural Reserve). The wet, scrubby habitat of the new species seen from the walkway beside the swamp. (Photograph by Huo Qing-Bo).

Figures 1 , 2 , 3 , 4 , 5 , 6 , 7

#### Type material.

***Holotype*,** 1♂, China, Guangxi Zhuang Autonomous Region, Guilin City, Ziyuan County, Maoershan National Natural Reserve, the walkway beside the swamp (Fig. 7), 1945 m, 25°53'37.2624"N, 110°25'25.1544"E, 27.VIII.2020, leg. Huo Qing-Bo (ICYZU). Paratypes, 1♂, 1♀, the same data as the holotype (ICYZU).

#### Diagnosis.

#### Description.

Adult habitus (Fig. 1): head black, antennae dark brown, pronotum dark brown with rugosities, head slightly wider than pronotum; cervical gills poorly developed, outside lateral cervical sclerites with single small membranous, gill-like nub. Wings subhyaline, infuscate, veins brown. Legs pale brown; abdominal segments brown, terminalia dark brown.

**Male** (Figs 2–4). Body length 7.5 mm, forewing length 9.0 mm, hindwing length 8.0 mm. Tergum VIII and tergum IX distinctly sclerotized, but median area is less sclerotized, distal margin of tergum IX slightly covering the anterior margin of tergum X, with a mid-anterior notch and a row of long spines extending to the anterior margin of tergum X along the posterior margin (Figs 2, 4). Hypoproct broad basally, and tapering to a thin apex; vesicle large, length approximately 2.5× width. Tergum X distinctly sclerotized at both edges of lateral area, mid-anterior area weaker, median area membranous, with two sclerotized triangular sclerite plates beneath epiproct; apex of sclerite strongly sclerotized and median portion with setae and several black spines, sclerotized band extending to lateral margin from sclerite base (Figs 2, 4). Cercus thick and oval, distinctly sclerotized, length approximately 2× width, gradually tapered toward tip with a long and slender spine, curving forward and inward at apex (Fig. 2A). Epiproct nearly oblong, short and broad; dorsal sclerite with two sclerotized bands forming a pair of nipple-like bulges, near apex with two S-shaped sclerotized arms tapering subapically toward small sharp tip, apex encased by cambered membrane with a small prolonged median sclerite (Fig. 2B, D); ventral sclerite with two stick-like sclerites bearing spines and connected at base forming a mid-posterior projection, basic sclerite sinuous with a lateral knob, partly extending posteriorly and upwards (Figs 3, 4). Paraproct divided into two lobes; outer lobe broad and short, strongly sclerotized with setae; inner lobe short, narrow at base, broader from 2/3 to apex with lateral margin strongly sclerotized (Figs 2–4).

**Female** (Figs 5, 6). Body length 9.0 mm, forewing length 10.5 mm, hindwing length 8.5 mm. Body coloration and the cervical gills are similar to the male (Fig. 5). Sternum VII definitely produced distally, extending to posterior margin of sternum VIII; pregenital plate rounded and wide, strongly sclerotized with several wrinkles. Sternum VIII with two obvious sclerotized spots and several small spots are dispersed laterally. Sternum IX and sternum X darkly sclerotized (Fig. 6). Paraproct dark brown and broad, cerci short and brownish.

#### Etymology.

The Latin *bi*- and *platta* referring to the paired sclerites present beneath epiproct.

#### Remarks.

Regarding to the affinities of the new species, *N. biplatta* belongs to the *cercispinosa* complex proposed by Baumann (1975), whose cerci enlarged and thick, bearing one or more spines at apex. The new species is similar to some of the species known from Assam like *N. khasii* Aubert, 1967 and *N. kuhleni* Aubert, 1967 by epiproct and paraproct. However, in *N. khasii* and *N. kuhleni*, the two lateral arms of the epiproct are rounder or heart-shaped, while in *N. biplatta*, the arms are sclerotized and S-shaped with a small sharp tip subapically. The outer lobe of paraproct differ quite slightly in shape. Compared to some Chinese species, the male of *N. biplatta* appears similar to *N. fusiformis* Chen & Du, 2017 and *N. nankinensis* Wu, 1926 from Jiangsu province, particularly in respect of the oblong epiproct. The epiproct ventral sclerite of *N. fusiformis* seems similar to our new species in the pair of prongs at the sides and the sclerotized lateral knob. However, the new species can be easily separated by the presence of the two sclerotized bands on the epiproct dorsal sclerite and the outer lobe of the paraproct without a strongly sclerotized large hook and a sharp process. In terms of *N. nankinensis*, the epiproct dorsal sclerite with a pair of lateral sclerites and the medially crossed grooves are quite similar to the new species, which may be distinguished by the pair of S-shaped sclerites with a sharp tip and the outer lobe of paraproct which is broad and blunt (without being finger-shaped) with a slightly curved apex. Above all, the sclerotized sclerite plate beneath the epiproct make it simple to identify it as a new species.

### Amphinemura cordiformis

Taxon classificationAnimaliaPlecopteraNemouridae

Li & Yang, 2006

BA78C775-D762-535E-A0FC-67976A4DB860

**Figure 8.**
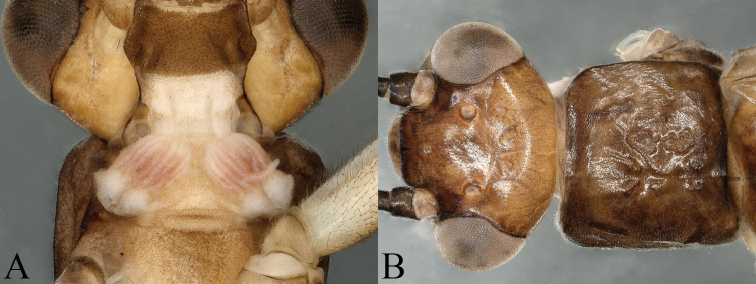
*Amphinemura cordiformis* male **A** prothorax, cervical gills, ventral view **B** head and pronotum, dorsal view.

**Figure 9.**
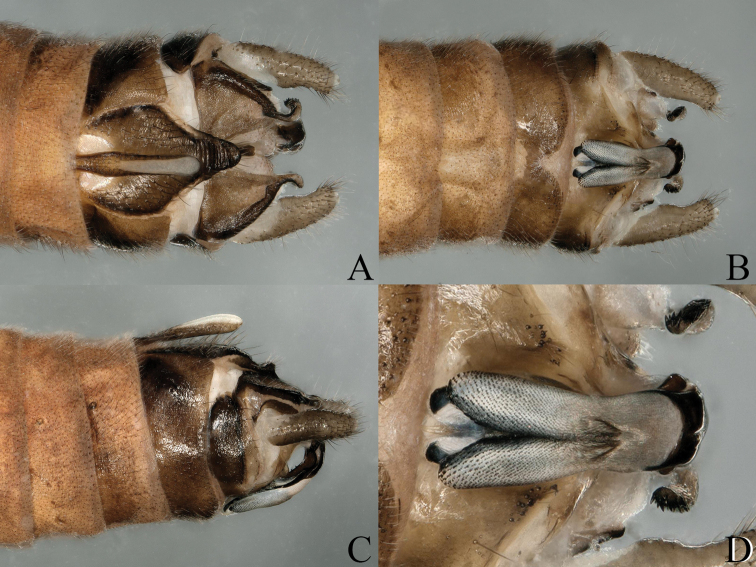
*Amphinemura cordiformis* male terminalia **A** ventral view **B** dorsal view **C** lateral view **D** epiproct, dorsal view.

**Figure 10.**
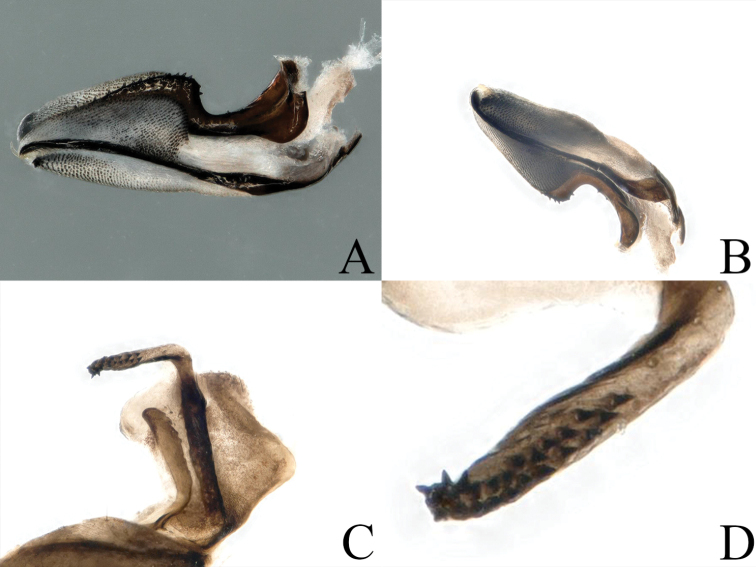
*Amphinemura cordiformis* male **A** epiproct, ventral view **B** epiproct, lateral view **C** right paraproct, ventral view **D** apex of the median right paraproct lobe, ventral view.

**Figure 11.**
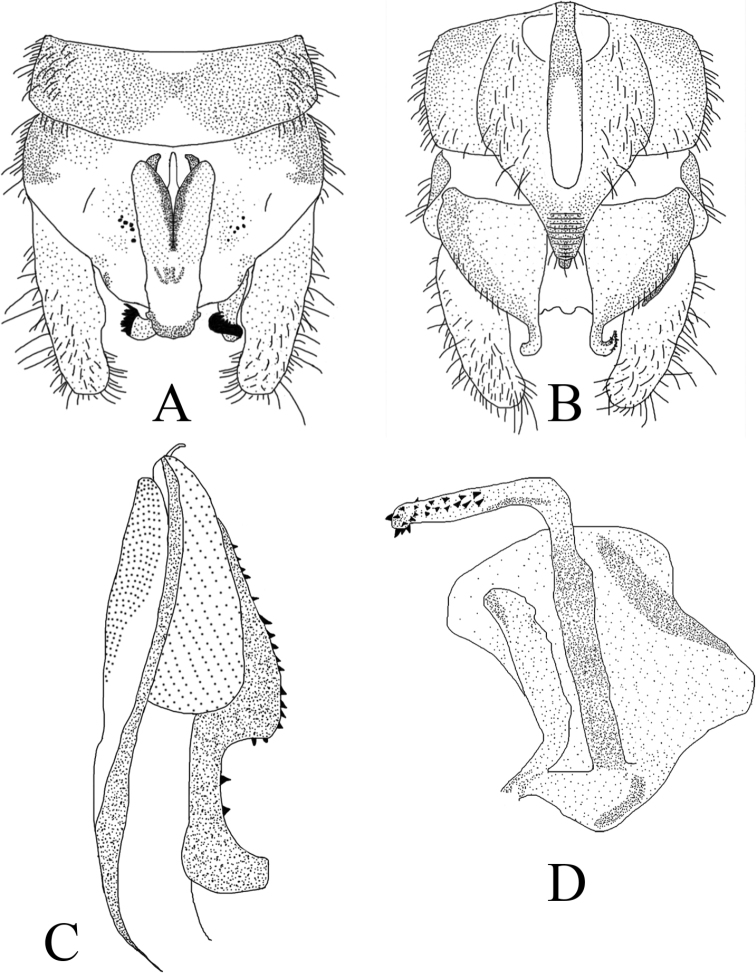
*Amphinemura cordiformis* male **A** terminalia, dorsal view **B** terminalia, ventral view **C** epiproct, lateral view **D** right paraproct, ventral view.

**Figure 12.**
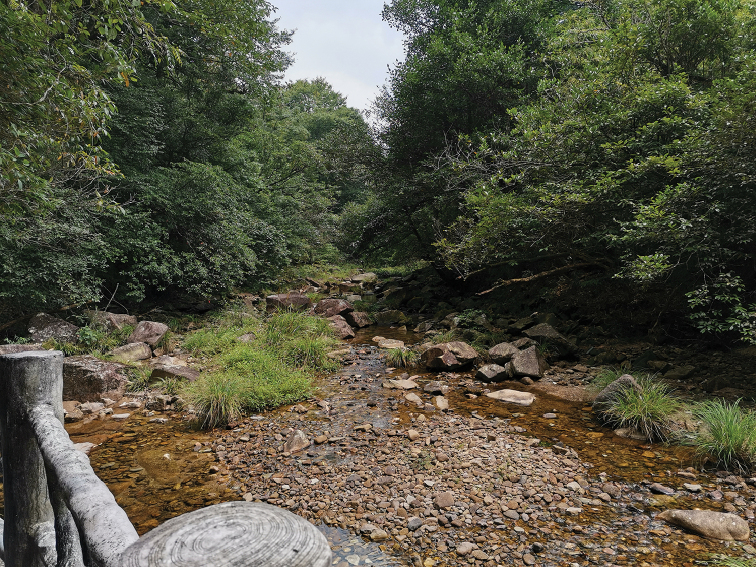
Habitat of *Amphinemura cordiformis* in China, Hunan Province, Mangshan National Natural Reserve, Guizizhai (Photograph by Huo Qing-Bo).

Figures 8 , 9 , 10 , 11 , 12

Amphinemura cordiformis : Li & Yang, 2006. Zootaxa 1154: 42.
Amphinemura cordiformis : Wang, Du, Sivec & Li, 2006. Illiesia 2(7): 50.
Amphinemura cordiformis : Yang, Li & Zhu, 2015. Fauna Sinica Insecta 58: 182.
Amphinemura cordiformis : Yang & Li, 2018. Species Catalogue of China. Vol. 2. Animals, Insecta (III), Plecoptera, 8.

#### Type locality.

China, Guizhou Province, Dashahe.

#### Material examined.

2♂♂, China, Hunan Province, Chenzhou City, Yizhang County, Mangshan National Natural Reserve, Guizizhai (Fig. 12), 1218 m, 24°57'4.896"N 112°55'44.418"E, 3.IX.2020, leg. Huo Qing-Bo (ICYZU); 12♂♂, China, Guizhou Province, Leigong Mountain, Lianhuaping, 1450–1620 m, 17–18.IX.2005, leg. Wang Zhi-Jie (ICYZU).

#### Distribution.

China (Guizhou, Hunan).

#### Diagnosis.

#### Description.

Adult habitus (Fig. 8): head and antennae dark brown, palpi pale brown, pronotum dark brown with rugosities, head wider than pronotum; two cervical gills, one on each side of lateral cervical sclerites with two branches, each branch divide into several branches; wing membranes subhyaline, veins brown. Legs pale brown; abdominal segments brown, terminalia darker.

**Male** (Figs 9–11). Body length 7.0 mm, forewing length 9.5 mm, hindwing length 7.2 mm. Tergum IX sclerotized with a concavity at mid-anterior margin, an inverted V-shaped concavity at mid-posterior margin (Figs 9B, 11A). Hypoproct broad basally, bearing setae and tapering to a thin apex, below apex with an apical nipple; vesicle slender, length approximately 4× of maximum width. Tergum X strongly sclerotized laterally, median area beneath epiproct weakly sclerotized with several black spots ambilaterally, covering sparse long setae (Figs 9, 11). Epiproct slender, split apically with a membranous small ligule; dorsal aspect of epiproct wrapped by two long, oval, apically grooved lobes, jointed at base and divided half-way by distinctly sclerotized along notch, below notch with a pair of V-shaped sclerotized stripes; dorsal sclerite with two slender, lateral sclerites projecting inwards to apex over lobes, forming two teeth-like tips; ventral sclerite entirely sclerotized, constricted basally with two small spines, broadened from half-way forming a subtriangular process with a row of black spines along margin, visible in lateral view; two subtriangular membranous lobes slightly shorter than process, located laterally, surface densely covered with pits (Figs 9–11). Paraproct trilobed; inner lobe weakly sclerotized, large and square, with slender sclerotized stripe along inner margin; median lobe mostly sclerotized, more strongly at base, subapically curved to form right angle, near apex two rows of small black spines, apex rounded with a ring of claw-like spines; outer lobe shorter than median lobe, weakly sclerotized, apex rounded and more heavily sclerotized, inner edge with some irregular nicks (Figs 10C, D, 11D).

#### Remarks.

Compared to the specimens described from Guizhou province, the ones from Hunan province present slight discrepancies in males. The Hunan specimens have a pair of V-shaped sclerotized stripes below the notch in the dorsal view of epiproct, and the ventral sclerite basally bears two small spines, which are obscure in the Guizhou specimens. Additionally, the paraproct outer lobe of the Hunan specimens is thicker, apically rounded, and bears some irregular nicks along the inner edge. The inner lobe has a slender sclerotized stripe along its inner margin and the median lobe bears two rows of spines subapically and a ring of claw-like spines apically whereas the inner lobe is triangular and slightly sclerotized in the Guizhou specimens, and the number and arrangement of the spines near the apex of median lobe are variable. As mentioned above, the enumerated characters probably refer to geographical or individual variability.

## Conclusion

The Nanling Mountains, where the two species discovered, including *Nemoura biplatta* sp. nov. from Maoershan National Natural Reserve in Guangxi Zhuang Autonomous Region and *Amphinemura cordiformis* from Mangshan National Natural Reserve in Hunan Province, are a priority area for biodiversity conservation. Although there are similar species exist, the two species documented here are new to science or represent new records based on detailed morphological comparison. Considering the geographical or individual variability of some similar species, molecular methods should be considered to confirm the status of new taxa in the future. Meanwhile, it is expected that more new species of stonefly may be discovered in the Nanling Mountains in the future with additional specimen collection and biodiversity surveys.

## Supplementary Material

XML Treatment for Nemoura biplatta

XML Treatment for Amphinemura cordiformis
